# Zinc Chloride Can Mitigate the Alterations in Metallothionein and Some Apoptotic Proteins Induced by Cadmium Chloride in Mice Hepatocytes: A Histological and Immunohistochemical Study

**DOI:** 10.1155/2023/2200539

**Published:** 2023-02-06

**Authors:** Enas Nihad Bayram, Nahla A. Al-Bakri, Hanady S. Al-Shmgani

**Affiliations:** Department of Biology, College of Education for Pure Sciences (Ibn Al-Haitham), University of Baghdad, Baghdad, Iraq

## Abstract

The heavy metal cadmium is extremely harmful to both humans and animals. Zinc supplementation protects the biological system and reduces cadmium-induced toxicity. This study aimed to determine whether zinc chloride (ZnCl_2_) could protect male mice with the damaged liver induced by cadmium chloride (CdCl_2_). The protective role of zinc chloride and expression of the metallothionein (MT), Ki-67, and Bcl-2 apoptotic proteins in hepatocytes were studied after subchronic exposure of mice to cadmium chloride for 21 days. Thirty male mice were randomly categorized into 6 groups (5 mice/group) as follows: a control group that did not receive any treatment, a group given ZnCl_2_ at 10 mg/kg alone, and two groups received ZnCl_2_ (10 mg/kg) in combination with CdCl_2_ at two concentrations (1.5 and 3 mg/kg), while the last two groups received CdCl_2_ alone at 1.5 and 3 mg/kg, respectively. Immunohistochemical examination revealed a decrease in Ki-67 expression in Kupffer and endothelial cells, which reflected cell proliferation downregulation accompanied by MT increased expression. However, the Bcl-2 was ameliorated and reduced to demonstrate an enhanced rate of necrosis rather than apoptosis. Furthermore, histopathological results showed significant alteration such as hepatocytes with a pyknotic nucleus, infiltration of inflammatory cells around the central vein, and the presence of many binucleated hepatocytes. Zinc chloride treatment resulted in histological and morphological improvements that were average in the expression of apoptosis proteins modifications induced by cadmium. Our findings revealed that the positive effects of zinc might be linked to the high metallothionein expression and enhanced cell proliferation. Furthermore, at low-dose exposure, cadmium-induced damage to cells could be more closely related to necrosis rather than apoptosis.

## 1. Introduction

Heavy metals are powerful biological toxins due to their toxicity, persistence, propensity to accumulate in organs, and food chain amplification [1]. Cadmium (Cd) is one of the most hazardous contaminants for the industry and the environment. The amount of cadmium in the air and in food chains has doubled in urban and industrial regions, posing a great risk to human and animal health. In addition, environmental and occupational exposure to cadmium has become a serious health problem in both developing and industrialized countries [2, 3]. Food and smoking are among the most significant causes of cadmium exposure to humans [4]. Wang et al. [5] reported that plant foods may contain higher extents of cadmium than meat, eggs, or milk in daily meals in their natural state depending on the extent of soil contamination. Ingestion of water or food, inhalation of particles, or inhalation of fumes during various industrial processes are the main ways that animals or humans are exposed to cadmium [6]. Continuous low-level exposure to Cadmium causes a progressive buildup in many human tissues, which has harmful effects on crucial organs such as the liver and kidneys [7]. Whether exposure was through ingestion, inhalation, or injection, the liver is the organ that will receive the most cadmium in the first hours following acute exposure, and following chronic exposure, the liver trails the kidney in the body's burden of cadmium as a result of the metal's bioaccumulation. This is because both the liver and kidneys have the capacity to produce metallothionein proteins. In our previous study, we reported that cadmium toxicity interferes with vital metals (zinc), induces oxidative stress, increases DNA strand damage, and triggers an active inflammatory response in the liver tissue [8]. Histological analysis is a critical and sensitive measure in identifying potential cellular alterations in the target organs exposed to heavy metals in order to determine its tissue pathology.

Zinc (Zn) is one of the most crucial trace elements in the body, involved in the biological processes of numerous proteins and enzymes. It is necessary for a variety of cellular functions, including cell division, immunity, and defense. Moreover, zinc is necessary as a trace intracellular element to combat free radicals but at a certain quantity, it could be harmful to most species [9].

MTs are low-molecular-weight proteins that are crucial for the detoxification of heavy metals (i.e., mercury and cadmium), the balance of vital metal ions (iron, zinc, and copper), and the prevention of oxidative damage by scavenging reactive oxygen species (ROS) [10]. The introduction of certain antioxidants should be a significant therapeutic strategy because oxidative stress is one of the key mechanisms of cadmium-induced damage. It has been observed that cadmium damages cells by producing ROS [11, 12]. The large bioaccumulation of cadmium in the liver and kidneys is attributed to their ability to produce high levels of MTs proteins, especially MT1 and MT2, which are found in the cytoplasm of cells, lysosomes, mitochondria, and nuclei. However, their construction depends on the presence of zinc and amino acids, especially the amino acid cysteine. MTs lead to the formation of Cd-MT complexes and thus contribute to cadmium retention in tissues and are responsible for the long biological half-life of cadmium in the body [13].

Ki-67 protein is a nuclear protein associated with the cell cycle. It is expressed in all phases of the cell cycle except the resting phase (G0) in quiescent cells or during DNA damage repair. Its half-life ranges from 60 to 90 minutes. The expression of the protein begins in the *S* phase of the cell cycle and gradually increases up to the G1 phase and then decreases after division [14, 15]. Baiomy and Mansour [16] indicated that there was a significant increase in the positive expression of Ki-67 protein in the hepatocytes of rats after oral treatment with drinking water containing cadmium chloride solution at 200 mg/kg.

Bcl-2 family proteins are pro- and antiapoptotic proteins that programmed the cell cycle and cell death signals. Bcl-2 is a member of the apoptosis regulatory protein, which is an antiapoptotic protein. Wang et al., in 2022, reported that cadmium chloride cytotoxicity in the human renal proximal tubule cells is related to mitochondrial respiration chain dysfunction leading to cell apoptosis by increasing ROS levels and decreasing Bcl-2 levels in a concentration-dependent manner. Also, mice exposed to cadmium showed different liver histopathological changes including increased apoptotic bodies, inflammatory cell infiltration, and hemorrhage [17].

Animal susceptibility to cadmium toxicity varied according to its concentration, but the cause of these variations is unknown and requires more investigation. As a consequence, the current study aimed to determine whether exposing mice to cadmium chloride would enhance apoptosis in the hepatocyte cells by elevating certain apoptotic proteins and whether zinc chloride might diminish or protect the tissue from damage. In addition, the relationship between the MT expression and liver pathologies was evaluated. We assumed that high MT expression induced by zinc chloride may lead to enhanced hepatic cell proliferation while decreasing apoptotic protein Bcl-2.

## 2. Materials and Methods

### 2.1. Chemicals

All chemicals and reagents were of analytical grade; zinc chloride (ZnCl_2_) and cadmium chloride (CdCl_2_) were purchased from Sigma-Aldrich (St Louis, MO, USA); Bcl-2, Ki-67, and metallothionein mouse specific HRP/DAB detection IHC kits were purchased from Abcam, UK. Hematoxylin and eosin (H&E) stain was provided by BDH Chemical Ltd., Poole, UK.

### 2.2. Animals

Thirty-six adult male mice (*Mus Musculus*) weighing 25 ± 5 g and aged 8–10 weeks were purchased from Animal House of Al-Nahrain University, Baghdad, Iraq. Mice were hosted at animal house laboratory in Ibn Al-Haitham College in an environment with controlled lighting (12–12 h light/dark) and temperature (25–5°C). Ad libitum access to water and food was provided.

### 2.3. Experimental Design

Mice were randomly chosen and divided into six groups of *n* = 6 mice/group. Group I, the control group, (GI) received sterilized distilled water. Group II (GII) received 10 mg/kg of ZnCl_2_ solution. Group III (GIII) received 1.5 mg/kg of CdCl_2_. Group IV (G IV) received ZnCl_2_ (10 mg/kg) + CdCl_2_ (1.5 mg/kg). Group V (G V) received 3 mg/kg CdCl_2_. Group VI (G VI) received ZnCl_2_ (10 mg/kg) + CdCl_2_ (3 mg/kg). All animals were injected intraperitoneally (IP) for 21 consecutive days, and at the end of the experiments, the mice were first anesthetized before being sacrificed by cervical dislocation. The University of Baghdad's Ethics Committee on Animal Use gave its approval to experimental methods no. D.A. 487 on 29 June, 2020.

### 2.4. Histopathological Examination

At the end of the experiments, the livers were dissected out and preserved in a 10% formaldehyde fixative, dehydrated in ascending grades of ethanol alcohol, cleared in xylene, and embedded in paraffin wax. For the histological analysis, thin sections (5 *μ*m) were cut and mounted on clean slides and then stained with hematoxylin-eosin (H&E) according to standard histological protocol [18]. Staining slides were examined and photographed using an Olympus microscope (BX41, Hamburg, Germany) with a digital camera.

### 2.5. Immunohistochemistry for the Detection of Ki-67, Bcl-2, and Metallothionein Proteins in the Liver Tissue

Liver samples embedded in paraffin wax (aforementioned histopathology section) were sections of 5 *μ*m thickness and loaded onto positive charged slides. Following dehydration and dewaxing, slides were dipped in an antigen retrieval solution, followed by blocking with a solution of 5% bovine serum albumin. Slides were incubated with primary antibodies for Ki-67 and Bcl-2 for an overnight period at 4°C. Slides were subsequently stained with hematoxylin and substrate chromogen solution after being treated with secondary antibodies at room temperature for two hours. The percentage of positive cells relative to a total of 100 cells in 10 slide fields served as the measurement for the expression of proteins of interest. The evaluation was completed in each case using five typical microscopic fields. With the aid of ImageJ software, the brown staining in liver cells was identified, and the positive staining area was measured in pixels. Two pathologists separately assessed the intensity of the immunohistochemical reactions [19, 20].

## 3. Results

### 3.1. Histopathological Study Results

**Figure 1:**
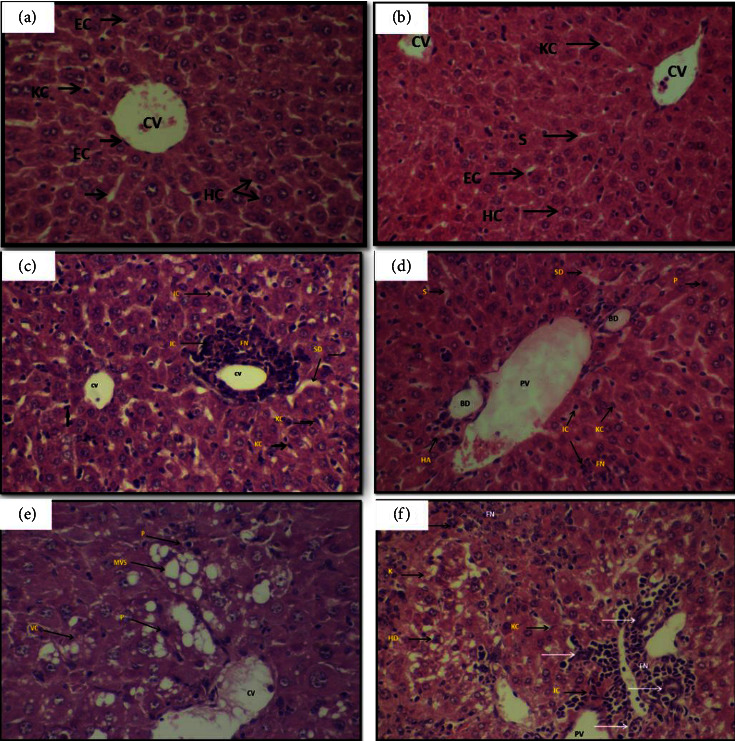
Liver section in mice showing the histopathological changes after exposure to cadmium chloride and/or zinc chloride. (a) Control group showing the normal tissue architecture, (b) treated with ZnCl_2_ 10 mg/k showing almost the normal structure cell, (c) treated with CdCl_2_ 1.5 mg/k, (d) treated with ZnCl_2_ 10 + CdCl_2_ 1.5 mg/k, (e) treated with CdCl_2_ 3 mg/k, and (f) treated with ZnCl_2_ 10 + CdCl_2_ 3 mg/k showing the central vein (CV), focal necrosis (FN), inflammatory cell (IC) infiltration, sinusoid dilation (SD), Kupffer cell(KC), signet ring cell (RC), portal vein (PV), bile duct (BD), hepatic artery (HA), sinusoide (S), pyknosis (P), macrovesicular steatosis (MVS), vacuolated cytoplasm (VC), karyolysis (K), hydropic degeneration (HD), and bile duct (BD). H&E stain, 40x.

The histological examination revealed a noticeable alteration to the liver due to the injection with various CdCl_2_ doses (1.5 and 3 mg/kg). The cadmium chloride treatment resulted in significant liver alterations, including defects in the radial arrangement of the hepatic cords, some hepatocytes with pyknotic nuclei and infiltration of inflammatory cells around the central vein with an assembly of red blood cells. Moreover, increased cadmium concentration (3 mg/kg) showed more pathological changes such as sever congestion in the portal vein and severe degenerative and necrotic changes of hepatocytes accompanied with Kupffer cells proliferation. Also, there were severe fatty changes in the liver compared to the previous group G3 (1.5 mg/kg) and control groups, where macrovesicular and microvesicular steatosis were observed in the cytoplasm of hepatocytes near the central vein, accompanied by the appearance of a pyknotic nucleus and vacuolated cytoplasm in other hepatocytes in the same region (Figure 1).

In contrast, ZnCl_2_ treatment at 10 mg/kg revealed ameliorated histological changes compared to cadmium treatment alone, where a clear decrease was observed in the appearance of focal necrosis areas with infiltration of inflammatory cells within the hepatic lobule around the vein. The central vein segment showed a similar appearance to the control group, and its endothelial lining could be distinguished, where the hepatic cords could be distinguished, and the sinusoids appeared among them, many of which appeared like the control, while some other sinuses, near the focal necrosis area, appeared dilated. In addition, hepatocyte degenerative changes seemed moderate compared to the changes recorded for the previous group G3 and were limited to the pyknosis of the nucleus, while no karyolysis was seen, and the cells did not show manifestations of fatty changes, and it was noted the presence of many binucleated hepatocytes (Figure 1). However, in the group that was treated with a higher dose of cadmium, there was less protective effect of zinc chloride, where hepatic sections showed severe congestion, increased necrotic area with infiltration of inflammatory cells within the hepatic lobule around the central vein, proliferation of Kupffer cells, and degenerative and necrotic changes in the hepatocytes at different stages within the same region, as many cells suffered from ballooning degeneration, hydropic degeneration, and complete cell necrosis (Figure 1).

### 3.2. Immunohistochemical Study Results

**Figure 2:**
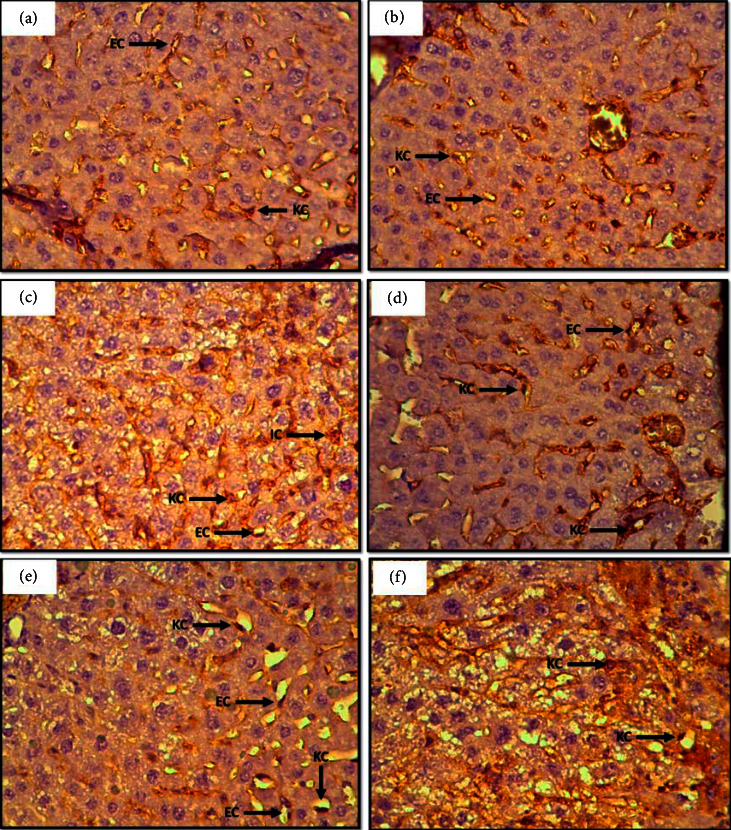
Liver section in mice showing immunohistochemical staining method detection of Ki-67 stained positive cells. (a) Control group showing a negative reaction in hepatocytes with weak positive stain in endothelial cells (EC) and Kupffer cells (KC). (b) Treated with ZnCl_2_ 10 mg/k showing almost similar reaction to control. (c) Treated with CdCl_2_ 1.5 mg/k the number of positive cells (brown stain) significantly increased. (d) Treated with ZnCl_2_ 10 + CdCl_2_ 1.5 mg/k. (e) Treated with CdCl_2_ 3 mg/k, the arrows show increased immunoreaction. (F) Treated with ZnCl_2_ 10 + CdCl_2_ 3 mg/k, 40x.

An assessment of the percentage of liver cells exhibiting a color reaction of the Ki-67 antigen is presented in Table 1 and Figure 2. Cells in the control group indicated a negative reaction in hepatocytes with weak positives in endothelial and Kupffer cells. The number of positive cells containing Ki-67 was increased in the Cadmium group compared to the control group. The ZnCl_2_ group revered less intensity expression in the G6 group than those in the G4 group (Figure 2). However, the percentage of positive staining is still higher than that of the control group (Table 1).

**Figure 3:**
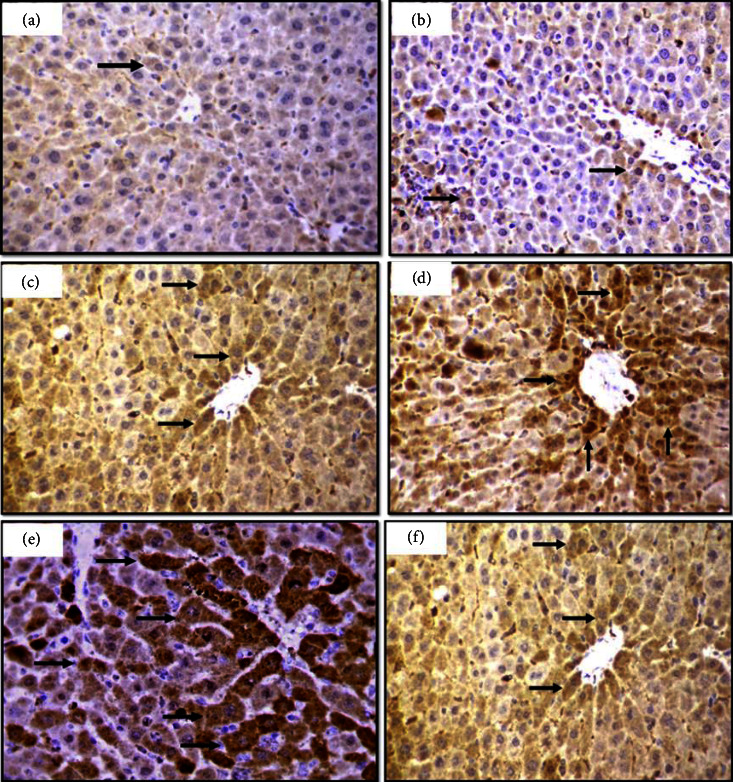
Liver section in mice showing immunohistochemical staining method detection of metallothionein (MT) stained positive cells. (a) The control group showing a few positive cells. (b) Treated with ZnCl_2_ 10 mg/k showing a slightly positive increased. (c) Treated with CdCl_2_ 1.5 mg/k. (d) Treated with ZnCl_2_ 10 + CdCl_2_ 1.5 mg/k, the positive cells are notably increased. (e) Treated with CdCl_2_ 3 mg/k. (f) Treated with ZnCl_2_ 10 + CdCl_2_ 3 mg/k showed a markedly increased immunoreaction, 40x.

The MT expression was weakly positive in the control group though there was a significant increase in cadmium groups (G3 and G4), and MT expression was further elevated in the G5 and G6 groups in a dose-dependent manner, as shown in Figure 3 and Table 2.

**Figure 4:**
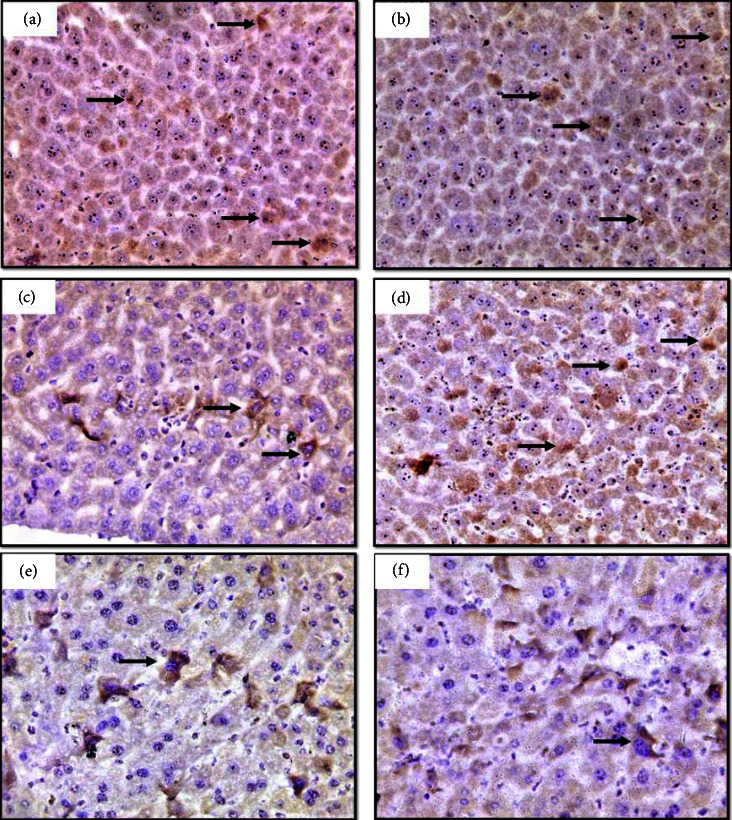
Liver section in mice showing immunohistochemical staining method detection of Bcl-2 stained positive cells. There are many positive cells (brown stain) in the control group (a), (b) treated with ZnCl_2_ 10 mg/k and (d) treated with ZnCl_2_ 10 + CdCl_2_ 1.5 mg/k groups. (c) Treated with CdCl_2_ 1.5 mg/k, there are few positive cells. (e) Treated with CdCl_2_ 3 mg/k and (f) treated with ZnCl_2_ 10 + CdCl_2_ 3 mg/k showed a decreased in immunoreaction, 40x.

Moreover, the Bcl-2 immunostaining percentage was decreased in groups cotreated with cadmium and Zn chloride compared to the control group; in contrast, ZnCl_2_ groups showed no obvious changes in the percentage of positive staining cells compared to the control group only after 21 days of exposure, as shown in Figure 4 and Table 3.

## 4. Discussion

In this study, we assumed that exposure of mice to cadmium chloride would promote apoptosis in hepatocyte cells by increasing certain apoptotic proteins and that zinc chloride could reduce or protect the tissue via increasing antioxidant proteins. Histopathology and immunohistochemistry of apoptosis in the liver revealed ameliorative zinc chloride protection. Zinc, a nutrient element, is believed to have a great effect on the metabolism and toxicity of cadmium due to its antioxidant properties [21]. Different mechanisms have been involved in the protection role of zinc in tissues; protective effects of Zn on thiols and other chemical groups, as well as its position as a structural component of the enzyme CuZn-superoxide dismutase (CuZn-SOD), all contribute to its ability to prevent damage from free radicals to biological structures [22]. Though, the current study's findings in the cotreatment (G4 and G6 groups) demonstrated a partial improvement in the liver tissue of male mice after 21 days of ip. Oxygen free radicals have been implicated in a number of studies as the cause of histological and cellular alterations [23]. In fact, these radicals can spread through membranes and into cells where they destroy biological components far from their original source. An imbalance in the synthesis of oxidants and antioxidants in the organ or organism is a sign of oxidative stress. Overproduction of ROS and reactive nitrogen species (RNS) or weakened oxidant defense mechanisms may both contribute to this imbalance [24]. According to the literature, subchronic oral exposure to cadmium causes numerous liver necrosis along with hepatic and ultrastructural alterations. Oxygen radicals are generated through the reduction of pathological alterations. The ability of zinc to induce metallothionein (MT), which is crucial for defense against Cadmium toxicity, could reduce the harmful effects of oxygen radicals [25].

Immunohistochemical results indicated that the Bcl-2 expression was decreased in group's exposure to cadmium. This finding along with the previous report [19] confirm that the cadmium-induced apoptosis may be linked to mitochondrial damage by upregulated expression of apoptotic mitochondrial proteins, such as members of the Bcl-2 family. This change in expression in Bcl-2 simultaneously activates caspase-3 and triggers apoptotic processes [26]. A significant increase in the caspase-3 protein level has been highlighted and reported in our previous study in mice treated with cadmium chloride, which resulted in increased apoptotic hepatocytes [8]. Moreover, the expression of apoptotic proteins is associated with increased levels of free radicals. It is acceptable to suggest that mitochondrial oxidative damage might take place causing the release of apoptotic proteins into the cytoplasm, which led to cellular death [27]. The capacity of a biological system to detoxify these reactive intermediates determines how much oxidative stress is experienced by cells on an ongoing basis [28]. Early in the reaction to oxidative stress, NF-E2-related nuclear factor 2 (Nrf2) is quickly elevated, and as a protective strategy, it then translocates to the nucleus to boost the transcription of oxidative stress response genes [29, 30].

Several studies suggested that Zn-induced metallothionein overexpression contributes to the Zn protective effect [29, 31]. In the present investigation, we discovered that Zn might, in a dose-dependent manner, cause MT overexpression. Thus, combining Zn and cadmium could partially inhibit Cadmium's cytotoxicity. The combined treatment with zinc and subsequently cadmium causes an increase in the production of MTs proteins in the liver of rats compared to the single treatment with cadmium alone, according to Khudhair and Abass [32]. The cotreatment in the G6 group, however, showed moderate improvement in liver tissues when compared to the G4 and G5 groups.

## 5. Conclusions

It is concluded that histopathological and immunohistochemical tissue analysis in mice livers injected with two subchronic doses of cadmium chloride revealed an increase in the death protein expression and decreased liver antioxidant protein (metallothionein). Conversely, cotreatment with zinc chloride at 10 mg/kg revealed an increase in the MT level expression in hepatic cells associated with Ki-67 expression in hepatocytes and Kupffer cells while decreased Bcl-2 expression and ameliorative histological alterations in the hepatic structure. The negative effects of cadmium can be partially reversed by increasing the synthesis of MTs proteins stimulated by zinc treatment, notably MT1. In addition, histological observations showed that necrosis was the main crucial mechanism for removing injured cells more than apoptosis in subchronic exposure to cadmium for a period less than a month of hepatotoxicity.

## Figures and Tables

**Table 1:** Ki-67 proliferation protein percentage in mice hepatocytes exposed to cadmium and zinc chloride calculated using ImageJ software.

Ki-67	Ki-67 proliferation protein (%), mean ± SE	*P* value	LSD
Control	11 ± 0.58	<0.001^*∗*^	6.78
Zn (10 mg)	12 ± 1.15		
Cd (1.5 mg)	21.5 ± 2.02^*ab*^		
Cd (1.5) + Zn (10)	15 ± 2.89		
Cd (3 mg)	35 ± 3.46^*abc*^		
Cd (3) + Zn (10)	37 ± 1.73^*abc*^		

^*a*^Significant differences vs. control group (*P* < 0.001), ^*b*^significant differences vs. all groups except G4, and ^*c*^significant differences vs. G4 group.

**Table 2:** Metallothionein protein percentage in mice hepatocytes exposed to cadmium and zinc chloride calculated using ImageJ software.

MT1	Metallothionein protein (%), mean ± SE	*P* value	LSD
Control	10 ± 1.15	<0.001^*∗*^	4.04
Zn (10 mg)	35 ± 0.58^*ab*^		
Cd (1.5 mg)	52 ± 1.15^*ab*^		
Cd (1.5) + Zn (10)	58 ± 1.15^*ab*^		
Cd (3 mg)	68 ± 1.73^*ab*^		
Cd (3) + Zn (10)	70 ± 1.73^*ab*^		

^*a*^Significant differences vs. the control group (*P* < 0.001) and ^*b*^significant differences vs. all other groups (*P* < 0.001).

**Table 3:** Bcl-2 protein percentage in mice hepatocytes exposed to cadmium and zinc chloride calculated using ImageJ software.

	Bcl-2 protein (%), mean ± SE	*P* value	LSD
Control	7.5 ± 0.29	<0.001^*∗*^	1.13
ZnCl_2_ (10 mg)	7.1 ± 0.35		
CdCl_2_ (1.5 mg)	5.4 ± 0.23^*ab*^		
CdCl_2_ (1.5) + ZnCl_2_ (10)	6.7 ± 0.40^*b*^		
CdCl_2_ (3 mg)	4.0 ± 0.58^*ab*^		
CdCl_2_ (3) + ZnCl_2_ (10)	3.6 ± 0.23^*ab*^		

^*a*^Significant differences vs. control (*P* < 0.001) and ^*b*^significant differences vs. all groups.

## Data Availability

The data used to support the findings of this study are included within the article.
